# Controlling skin microbiome as a new bacteriotherapy for inflammatory skin diseases

**DOI:** 10.1186/s41232-022-00212-y

**Published:** 2022-09-01

**Authors:** Yoshihiro Ito, Masayuki Amagai

**Affiliations:** 1 Department of Dermatology, Keio University School of Medicine, 35 Shinanomachi, Shinjuku-ku, Tokyo, 160-8582 Japan; 2 RIKEN Center for Integrative Medical Sciences (IMS), 1-7-22 Suehiro-cho, Tsurumi-ku, Yokohama, Kanagawa 230-0045 Japan

**Keywords:** Skin microbiome, Atopic dermatitis, Acne vulgaris, Inflammatory skin disease, Microbiome composition analysis, Bacteriotherapy, Bacteriophage

## Abstract

The skin serves as the interface between the human body and the environment and interacts with the microbial community. The skin microbiota consists of microorganisms, such as bacteria, fungi, mites, and viruses, and they fluctuate depending on the microenvironment defined by anatomical location and physiological function. The balance of interactions between the host and microbiota plays a pivotal role in the orchestration of skin homeostasis; however, the disturbance of the balance due to an alteration in the microbial communities, namely, dysbiosis, leads to various skin disorders. Recent developments in sequencing technology have provided new insights into the structure and function of skin microbial communities. Based on high-throughput sequencing analysis, a growing body of evidence indicates that a new treatment using live bacteria, termed bacteriotherapy, is a feasible therapeutic option for cutaneous diseases caused by dysbiosis. In particular, the administration of specific bacterial strains has been investigated as an exclusionary treatment strategy against pathogens associated with chronic skin disorders, whereas the safety, efficacy, and sustainability of this therapeutic approach using isolated live bacteria need to be further explored. In this review, we summarize our current understanding of the skin microbiota, as well as therapeutic strategies using characterized strains of live bacteria for skin inflammatory diseases. The ecosystem formed by interactions between the host and skin microbial consortium is still largely unexplored; however, advances in our understanding of the function of the skin microbiota at the strain level will lead to the development of new therapeutic methods.

## Introduction

The skin is the outermost barrier that divides life from the external environment. In addition to skin functioning as physical and chemical barriers, the skin provides habitats for complex microbial community termed “he skin is the including trillions of bacteria, fungi, mites, and viruses interacting with one another [1]. Similar to the microbial flora of the intestinal tract, the skin microbiota play an essential role in defense against invading pathogens and in the education of immune system [2, 3]. The environment for skin microbiota is defined by the anatomical site and skin microenvironments, including lipids, salts, pH, peptides, and water, which correlate with the composition of these distinct microbial communities [4, 5]. Although the skin is constantly affected by external environmental factors and acquires a large amount of transient microbiota, the composition of skin microbial communities is largely stable over time [6–8].

An imbalance in host-microbe interactions affected by endogenous (e.g., age or genetic variation) or exogenous (e.g., antibiotics or soap) factors can result in skin disorders or infections. Changes in composition of microbial community during disease are referred to as dysbiosis [9, 10]. Pronounced examples of dysbiosis include a decrease in diversity at the phylum level, as observed in atopic dermatitis (AD) [11–13], rosacea [14–16], and acne [17, 18].

Recent insights into the skin microbiome have been applied to new therapeutic strategies using live bacteria, termed bacteriotherapy, based on an understanding of how bacteria communicate with the host and other skin microbiota. In this review, we discuss the interactions within this ecosystem and the possibility of new therapeutic strategies using skin microbiota to maintain homeostasis and prevent skin inflammatory diseases.

### Skin physiology and microbial topography

**Fig. 1:**
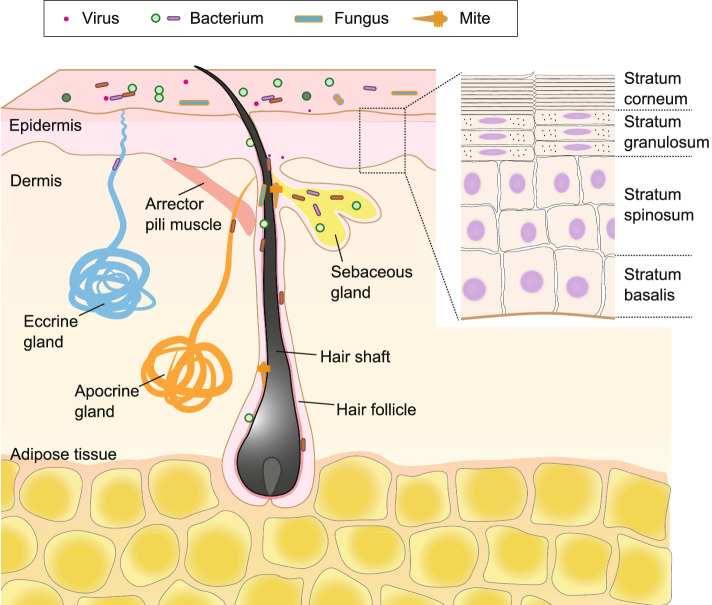
Structure of the skin and microbiota. The skin is the outermost barrier that covers the entire body and provides habitats for the microbiota (viruses, bacteria, fungi, and mites). The skin consists of epidermis, dermis, and adipose tissue. The epidermis is divided into four layers: stratum basalis, stratum spinosum, stratum granulosum, and stratum corneum, from the basal side to the apical side. The dermis and adipose tissue contain skin appendages, such as sweat glands (eccrine and apocrine glands), hair follicles, hair shafts, sebaceous glands, and arrector pili muscles. Eccrine glands produce sweat and open directly onto the epidermal surface through the duct, whereas apocrine glands and sebaceous glands connect to the hair follicle. The skin microbiota colonizes both the surface and appendages, and its composition varies depending on the microenvironment

The skin is composed of three distinct layers: epidermis, dermis, and adipose tissue (Fig. 1). The epidermis, the outermost layer, is composed of differentiated keratinocytes. Epidermal keratinocytes undergo differentiation from mitotically active basal cells (stratum basalis) to spinous cells (stratum spinosum) to granular cells (stratum granulosum), preparing for the dissolution of the nucleus and organelles. This results in the stratum corneum, which is the top layer composed of finally differentiated and enucleated squames (corneocytes) that are chemically cross-linked to strengthen the skin barrier [19]. The stratum corneum consists of approximately 10 layers of corneocytes containing keratin fibrils and cornified envelopes embedded in lipid bilayers, forming the “bricks and mortar” of the epidermis [19]. However, a recent study revealed that the stratum corneum is formed by a meticulous mechanism regulated by a prolonged calcium surge and pH change in the cytosol, resulting in the degradation of organelles in the stratum granulosum layer [20]. This specific type of functional cell death, called corneoptosis, in which remnants of dead keratinocytes remain functional in the stratum corneum, is unique and essential for the maintenance of skin barrier function [20]. Physiologically or genetically determined variations in the stratum corneum properties lead to dysbiosis, which is a cause or a result of agitation of the skin barrier function and exacerbation of chronic inflammatory skin diseases such as AD, psoriasis, and acne [12, 21–24].

In addition to the physiological functions of maintaining homeostasis, skin appendages are relevant interfaces for host-microbe interactions [25]. Sweat glands are divided into two types. Eccrine glands, which are more abundant than apocrine glands, are distributed across most skin surfaces and stabilize body temperature by providing secretion, which mainly contains water and salt [26]. Apocrine glands are found in certain regions of the body such as the armpits, beards, nipples, and some parts of the external genitalia. Apocrine glands periodically secrete a viscous, odorous product, and the excretory ducts open into hair follicles (HFs) [27, 28]. Sweat glands evaporate water for thermoregulation and acidification of the skin, resulting in unfavorable conditions for bacterial growth. Moreover, free fatty acids and wide-ranging antimicrobial peptides (AMPs), such as dermcidin [29] and cathelicidin [30], are secreted from sweat glands to inhibit the growth of various microbes, resulting in a change in the composition of the microbial community [31, 32].

HFs and hair shafts offer broad functionalities, including thermoregulation, physical protection from environmental stimuli, activity as a sensory organ, and facilitating interactions with microbes [33]. One of the main evolutionary functions of HF is to provide a habitat for the skin microbial community and generate an interface for microbial-epithelial interactions [34]. Moreover, the immune system matures through host-microbe interactions in HF [35–37]. Compared with the skin surface, the HF epithelium provides a moist, well-perfused, and relatively ultraviolet light-protected tissue column under aerobic conditions, which is favorable for bacterial growth [34]. These unique habitats in the HF facilitate the colonization of bacteria, fungi, and viruses [38–40].

Sebaceous glands produce and secrete sebum, which contains complex oils, such as triglycerides, fatty acid degradation products, wax esters, squalene, cholesterol esters, and cholesterol [41]. Sebaceous glands connect to HF and secrete sebum, which provides the hydrophobic coat of the hair and skin that acidifies the surface and functions as an antibacterial shield and moisturizer [32]. Alternatively, sebum is a source of lipids used as a nutrient source by specific commensals, such as *Cutibacterium acnes* (formerly *Propionibacterium acnes*), which produce lipases and utilize the decomposed fatty acids. The lipids of sebum are also metabolized by several skin microbiota, such as *Malassezia* and *Corynebacterium* spp., which are unable to produce their own lipids [42]. HF, hair shaft, associated sebaceous gland, and arrector pili muscle are collectively known as pilosebaceous units.

The skin is an important organ for adrenal glucocorticoid synthesis. Human keratinocytes are capable of de novo extra-adrenal cortisol and sex hormone synthesis, which may be a fundamental pathway for skin homeostasis [43–45]. Additionally, major skin compartments such as the epidermis, dermis, and HFs have been shown to synthesize cortisol, an endogenous glucocorticoid that is administered as a potent anti-inflammatory drug for chronic inflammatory diseases [46, 47]. Several types of cells in the skin such as keratinocytes, dermal fibroblasts, and sebocytes express 11β-hydroxysteroid dehydrogenases (11β-HSDs), which modulate the availability of cortisol in peripheral tissues by interconverting its active and inactive forms [48]. The expression of 11β-HSD1, which activates cortisol, is decreased in patients with AD [49, 50]. Similarly, local inhibition of 11β-HSD1 exacerbated inflammation in a mouse model of AD [50]. Furthermore, we recently revealed that colonization with a specific strain of commensal skin microbe promotes local glucocorticoid synthesis [51]. These findings suggest that endogenous local glucocorticoid synthesis is mediated by host-microbe interactions and is important for maintaining homeostatic conditions.

The body’s anatomical sites provide diverse microenvironments that vary in humidity, lipids, temperature, pH, and sebum content [1]. Skin habitats are mainly composed of the stratum corneum and skin appendages such as HFs, sweat glands, and sebaceous glands [1]. These dermal appendages are associated with their unique microbial communities owing to their physiological characteristics [52, 53]. Moreover, microbial components are found on the entire surface area of the skin appendage and even below the basement membrane, including in the adipose tissue [54, 55]. Based on these characteristics and the composition of the skin microbiome, body sites are divided into three anatomical positions: oily, moist, and dry [1, 2, 32]. The environment of these parts is influenced by the abundance and activity of skin appendages [32]. Oily sites such as the forehead, chest, and back, where sebaceous glands are dense and active, are dominantly colonized by lipophilic *Cutibacterium* and *Staphylococcus* genera [56]. Generally, the bacterial diversity of sebaceous sites seems to be low, suggesting that microenvironments containing rich sebum select specific subsets of microorganisms that can tolerate the conditions in these areas [1]. Alternatively, moist sites such as the elbow (antecubital fossa) and knee (popliteal fossa), where sweat glands are more abundant, were preferentially dominated by bacteria that thrive in humid environments, such as *Corynebacterium* and *Staphylococcus* genera [4]. Dry sites, such as the volar forearm and palm, harbor diverse microbial communities, including members of *Actinobacteria*, *Proteobacteria*, *Firmicutes*, and *Bacteroidetes* genera [57, 58]. More specifically, *Cutibacterium*, *Corynebacterium*, and *Streptococcus* species predominantly colonize dry sites [32]. Compared with other skin sites, dry sites experience large fluctuations in surface temperature. Culture-based methods have revealed that these areas have quantitatively lower biomass than the skin surface of moist areas. Compared with the bacterial community, the biomass of fungi and viruses is relatively lower at body sites [58]. Fungi of the genus *Malassezia* are dominant at the core body and arm sites, whereas foot sites show high fungal diversity, including members of *Malassezia*, *Aspergillus*, *Cryptococcus*, *Rhodotorula*, *and Epicoccum* genera [58, 59]. In contrast to bacteria and fungi, eukaryotic DNA viruses detected in the skin are unique to the individual rather than site specific [6]. *Cutibacterium* and *Staphylococcus* bacteriophages are predominant within the viral fraction of the skin, suggesting that bacteriophages contribute to modulating bacterial populations [6, 60]. Additionally, other double-stranded DNA viruses, such as human papillomaviruses, *Merkel cell polyomavirus*, and *Molluscum contagiosum virus*, which are associated with dermatological lesions, were detected in about half of the subjects in a study using shotgun metagenomic sequencing [6].

### Microbiome composition analysis

Improvements in high-throughput microbial genomic sequencing analysis techniques, including amplicon sequencing and whole-genome sequencing (shotgun metagenomic sequencing), have made it possible to reveal not only the composition but also the function of skin microbial communities. Traditionally, skin microbial members have been investigated using culture-based methods. This approach selects only specific culturable microorganisms that thrive under artificial growth conditions. Therefore, the total community diversity was underestimated. Culture-independent metagenomic approaches using next-generation sequencing have increased the sensitivity and power of associative studies to avoid the bias introduced by the use of culture and capture the complete diversity of the microbiome [61]. In these high-throughput analyses, the 16S ribosomal RNA (rRNA) gene or internal transcribed spacer 1 (ITS1) region has been used in multiple studies of skin microbial communities to identify bacteria and fungi, respectively [62, 63].

With technological advances from Sanger sequencing to next-generation sequencing, these original approaches are constantly refined to accommodate increased read depth and shorter read length. This development has been achieved with new primers and clustering methods to overcome sequencing errors and assembly methods to combine paired-end reads. A subregion of the 16S rRNA gene was chosen for amplification and analysis with shorter amplicon lengths (approximately 300–400 bp compared to > 1000 bp in Sanger sequencing). Bacterial 16S rRNA contains nine hypervariable region (V1-V9); for instance, the V1-V3 or V3-V4 region is generally used for skin microbial composition analysis with shorter read lengths. The amplicons of hypervariable regions can be used to classify each amplicon at the relative abundance of the genus and, when possible, at the species level. However, the resolution of sequencing analysis depends on the region of the 16S rRNA gene and the microbial community to be analyzed. For instance, sequencing of the V4 region poorly captures several commensal skin microbiota, especially *Cutibacterium* [64]. More recently, the whole region of 16S rRNA has been used for several years to analyze the microbial diversity at the species level using long-read sequencing platforms such as PacBio or Nanopore [65].

While amplicon sequencing targeting specific regions of 16S rRNA only provides insight into the taxonomic composition of the bacterial community, shotgun metagenomic sequencing allows for higher-resolution analysis of species-, strain-, and single-nucleotide variant-level diversification and community functions that are associated with skin microbiota [1, 66]. In the preparation of the shotgun metagenomic sequencing method, small fragments of DNA extracted from all cells in a community are independently sequenced, resulting in DNA sequences that align to various genomic locations for the genomes of multi-kingdom microbiota coexisting in the sample [67].

Although methods for analyzing the composition and function of microbial communities are highly informative, current methods using microbial DNA have limitations. For instance, many important phenomena by host-microbe and/or microbe-microbe interactions occur at the strain level, whereas sequencing approaches reveal species-level taxonomic components at maximum resolution. Similarly, the majority of models for microbiome study design do not involve longitudinal sampling, resulting in failure to capture the dynamic behavior of microbial communities [67]. Additionally, sequence-based microbiome studies support the notion that commensal bacteria correlate with human health and disease; however, they do not address the causality and direction of host-microbe interactions, and disease-related dysbiosis can reflect only a tiny fraction of the host pathophysiology [68]. To address these limitations, causative research is essential for the development of therapeutic agents that focus on interactions between the host and microbes. In other words, animal models relevant to the disease are required to be utilized to explore the influence and mechanistic role of microorganisms isolated from patients or healthy individual [32].

Several methods have been used to collect skin microbiota for amplicon analysis or shotgun metagenomic analysis, such as swabbing, biopsy, surface scraping, or tape strip. Since each skin commensal inhabits different depths or skin appendages, the microbial community in the sample depends on the method used to collect them [69–72]. Microbial compositional analysis showed comparable results regardless of the sampling method [73, 74]; however, these analyses lost the location information of each skin microorganism. Advances in sequencing technology have made it possible to analyze the compositional and functional analysis of microbial communities at high resolution, and in the future, host-microbe interactions in the microenvironment of the skin need to be elucidated in order to understand the full picture of the crosstalk between host and microbiota. This spatial information may be of great help in elucidating the microenvironment for host-microbe interactions.

### Role of the skin microbiome

#### Healthy skin microbiota

While the crosstalk between the host and individual skin microbes remains largely unclear, their function as a consortium is important for maintaining skin homeostasis. The skin microbiota is divided into two groups: resident microorganisms (core microbiota) that are stably colonized on the skin, and the others are transiently living microbes from the external environment that persists for hours to days before disappearing [75]. The core microbiota are stable over time despite external exposure [6] and are considered harmless to the host, providing some benefit to each other. Under normal conditions, the transient skin microbiota also appears to be nonpathogenic [76]. Microbial protease enzymes are involved in the desquamation process in the stratum corneum, and degradation of sebum and free fatty acids by microbiota contributes to pH regulation on the skin surface [60, 77]. Besides, the microbial barrier acts as a shield that protects the body against potential pathogens by competition [72] and AMP production [78]. Host-microbe crosstalk contributes to maintaining the barrier integrity by activating keratinocyte aryl hydrocarbon receptor [79] and maturation of skin barrier functions, including education of the immune system [77, 80], wound repair [81–85], and protection against skin cancer [86].

Certain strains of *Staphylococcus epidermidis*, the most prominent skin commensal, benefit the host by activating the host innate immune response against pathogens via toll-like receptor (TLR) 2 [1, 87], producing AMPs and phenol-soluble modulins (PSMs) against pathogens, such as *S. aureus* and *Streptococcus* spp., and stimulating the production of AMP from the host [88–90]. Proteases from *S. epidermidis* inhibit biofilm formation and eliminate colonization by pathogenic bacteria [91, 92]. Additionally, colonization with *S. epidermidis* accelerates wound closure by recruiting neutrophils and activating type I interferon (IFN)-producing plasmacytoid dendritic cells [85]. Other skin commensals, such as *Staphylococcus lugdunensis* and *Staphylococcus hominis*, also inhibit *S. aureus* growth through the production of the antibiotic lugdunin and lantibiotics, respectively [93] [78].

Collectively, it is expected that interactions of single or several bacterial strains with the host will be further elucidated using high-throughput sequencing technology and in vitro and in vivo experimental models.

#### Pathogenic skin microbiota

Previous studies of the skin microbiome have mainly targeted disease-associated commensal bacteria. The balance of interplay between the host and bacterial population is continuously affected by intrinsic (such as genetics, hormones, and aging) and extrinsic (such as lifestyle, geographical location, and usage of antibiotics) factors that change the composition of the microbial community and host skin barrier function [76]. Accordingly, the composition of the cutaneous microbial community can shift depending on disease progression [94]. Additionally, specific commensals have been implicated in several inflammatory skin diseases. Using genomic and metagenomic approaches, the roles of microbial components that stimulate or modulate host responses have been proposed. *Staphylococcus aureus*, the most well-known pathogenic skin microbiota, is associated with AD severity. AD is a chronic inflammatory skin condition that is clinically characterized by relapsing eczematous lesions, pruritus, and a fluctuating course and pathogenically by defective skin barrier, dysbiosis, and type-2 immune responses [95]. In AD skin lesions, overgrowth of *S. aureus* and a decrease in the overall microbial diversity are remarkable findings [11]. *S. aureus* caused eczematous dermatitis in a genetically modified mouse model of AD [51, 96]. Moreover, toxins, such as δ-toxin [97], α-toxin [98], and PSM α [99, 100], promote both innate and adaptive immune responses that elicit skin inflammation. *S. aureus* occasionally induce life-threatening infections such as endocarditis [101], skin and soft tissue infection [102, 103], osteomyelitis [104, 105], toxic shock syndrome [106, 107], and food poisoning [108]. This opportunistic pathogen *S. aureus* asymptomatically colonizes more than 30% of healthy individuals in relatively low and barely detectable abundance levels under steady state [69, 109–111], indicating that additional host or environmental factors or potentially pathogenic bacteria are required to elicit skin inflammation.

Another example is acne vulgaris, in which *C. acnes* is implicated as a pathogenic factor. *C. acnes* produces various pathogenic factors, including proteases, lipases, and chemotactic factors for neutrophils, macrophages, and lymphocytes [112]. Acne vulgaris is a common skin disease characterized by chronic inflammation of pilosebaceous units associated with bacterial colonization of HF on sebaceous sites by *C. acnes*, androgen-induced increase in sebum production, altered keratinization, and inflammation [113, 114]. The skin of patients with acne and healthy individuals showed that the strain population structures of *C. acnes* were significantly different, whereas the relative abundances were not different [24]. Metagenomic analysis of these strains revealed potential pathogenic factors that have not been previously reported, indicating the importance of high-resolution analysis at the strain level to unveil the pathogenesis of human diseases [24, 115]. In addition to acne, *C. acnes* is involved in various diseases such as implant-associated infection [116, 117], prostate inflammation [118], and sarcoidosis [119, 120].

Several species of *Corynebacterium*, one of the dominant genera on the skin, have been proven to be pathogenic. Dysbiosis is characterized by dominant colonization and overgrowth of *Corynebacterium mastitidis* and *Corynebacterium bovis* and is associated with the development of AD features in a mouse model [96]. *Corynebacterium accolens* promotes skin inflammation in an IL-23-dependent manner, and mycolic acid, which is a major component of the *Corynebacterium* cell wall, is essential for mediating these responses in mice [121]. Alternatively, antibacterial free fatty acids produced from skin surface triacylglycerols by *C. accolens* inhibit the growth of *Streptococcus pneumoniae*, a potentially pathogenic bacterium that causes pneumonia, septicemia, and meningitis [122]. Similarly, certain strains of *S. epidermidis* opportunistically demonstrate pathogenicity even though they potentially benefit the host [123, 124]. These findings suggest that skin microbiota are beneficial to the host in some aspects; however, under certain circumstances, they exhibit pathogenicity by causing inflammation.

### New therapy using skin microbiota (bacteriotherapy)

The role of the skin microbial community in maintaining homeostasis and its involvement in diseases is gradually being elucidated, contributing to our understanding of the skin ecosystem [2]. Although bacteriotherapy for skin diseases is a challenging field, the concept of manipulating the composition and function of the skin microbiota to improve disease state has been recently investigated through the elucidation of the skin ecosystem [125].

Although antibiotics are commonly used to target colonized bacteria to treat infections or inflammatory diseases, there are risks that must be considered. Oral or topical antibiotics are also widely prescribed in the field of dermatology because of the high frequency and chronicity of cutaneous diseases associated with skin commensals, such as acne. However, systematic studies focusing on the effect of antibiotics on the skin microbiome and the pervasiveness of these effects are lacking. The administration of antibiotics systemically affects the composition of the microbiome, including the gut and skin, and commensals beneficial to the host may be eliminated [126]. Alterations in the microbial community disturbed by antibiotics can be expected to affect host physiology and potentially host health, including increased susceptibility to diseases such as psoriasis (evaluated in mice), AD, and asthma (evaluated in human) [127–131]. Furthermore, prolonged administration of antibiotics is fraught with the risk of infections caused by multidrug-resistant bacteria, which can be life-threatening in some immunosuppressed patients [132]. Therefore, a new therapeutic method that eliminates specific pathogens would be ideal.

There are two strategies of treatment using live bacteria for skin diseases. The first is to colonize a bacterial consortium that ameliorates the ecology of the skin. The second targets a specific pathogen by producing AMP or other metabolites from the reintroduced living microbiota or host cells stimulated by transplanted bacteria. Thus far, the latter has been investigated more in the field of dermatology.

**Fig. 2:**
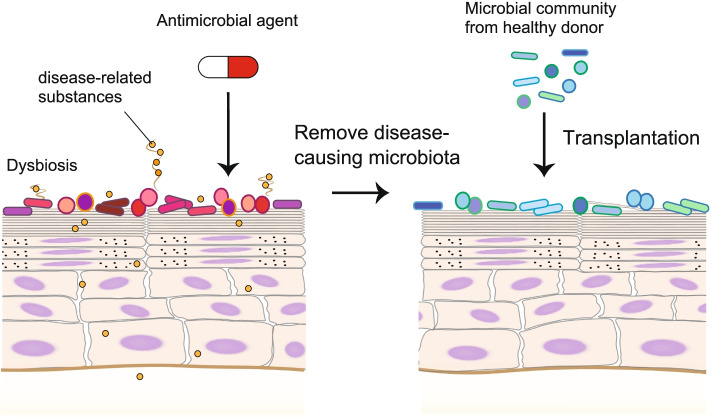
Transplantation of skin microbial community. One therapeutic strategy using live bacteria for skin diseases is transplantation of the whole skin microbial community from healthy donors to diseased skin associated with dysbiosis, such as patients with malodor. Pre-colonized disease-causing skin microbiota are removed by antibacterial agents such as antibiotics. Subsequently, the microbial community collected from the skin of non-odorous donors is transplanted to the patient’s skin to occupy space and provide nutrition, preventing other bacteria from colonizing the skin niche and producing disease-causing substances

Similar to the treatment of diseases of the intestinal tract with bacterial communities, the treatment of skin diseases with microbial transplantation is under development and could provide a promising approach for the treatment of cutaneous diseases [133, 134]. Transplantation of the skin microbial community to correct unsuitable armpit odors is currently a possibility under consideration [60]. The malodor-causing microbiota was removed by antibacterial agents and replaced with a skin microbial community collected from a non-odorous donor (Fig. 2). An important point to be addressed is whether the transplanted bacteria can stably colonize the skin. Simply transferring skin microbiota is insufficient for colonization [135]; thus, optimized transplantation strategies, including administration methods and antibacterial agents to open the niches, are required. A prominent example of the possible efficacy of microbial transplantation is fecal microbial transplantation (FMT) for *Clostridium difficile*-associated colitis. Transplanted gut microbial communities from healthy donors showed success rates of over 80% in treating infections with *C. difficile*, a nosocomial pathogen often recalcitrant to antibiotics [136, 137]. Although synthetic reproduction of the complex ecosystem with microbial transplantation remains a powerful therapeutic option, the US Food and Drug Administration (FDA) has not yet approved FMT for *C. difficile* and alerts the potential risk of serious or life-threatening infections with transplanted bacteria [138]. To gain the approval of FDA, it is necessary to clarify the components of the bacterial consortium and characterize each member to be administered. Furthermore, mining and optimizing a single strain or combination of microbiota should be considered to achieve the maximum effect in a stable manner [68].

**Fig. 3:**
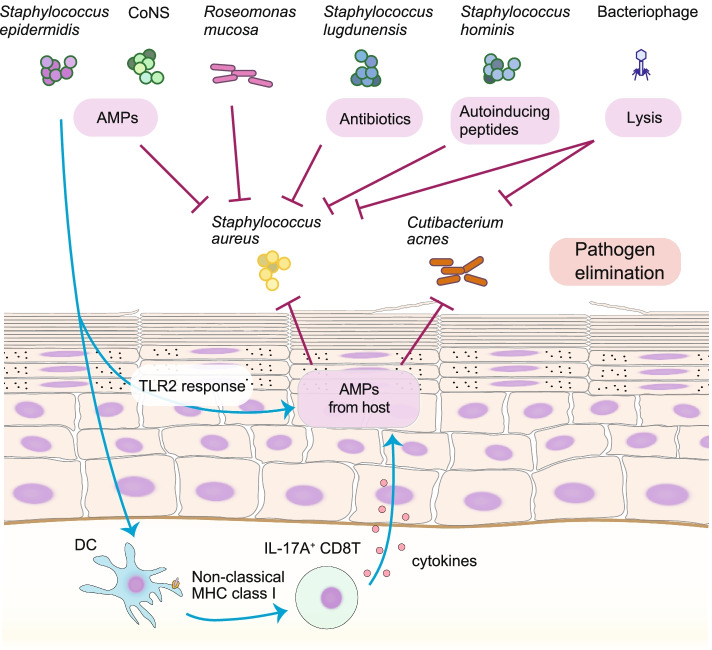
Potentially therapeutic skin microbiota by pathogen elimination. Engraftment of specific skin microbiota that play a role in killing the pathogen is a therapeutic strategy for skin inflammatory diseases such as atopic dermatitis and acne. Colonization of *Staphylococcus aureus* is inhibited by bacterial metabolites such as antimicrobial peptides (AMPs), autoinducing peptides, and antibiotics. Bacteriophages kill and lyse the bacteria that they infect. *Staphylococcus epidermidis* promotes the production of AMPs from host cells through the Toll-like receptor 2 (TLR2) response. Dendritic cells (DCs) capture *S. epidermidis* and induce IL-17A+ CD8T cells via a nonclassical MHC I-restricted pathway. Induced IL-17A+ CD8T cells produce cytokines that stimulate keratinocytes to produce AMPs against pathogens

Another treatment strategy for skin diseases is the use of a characterized strain of skin microbiota that suppresses disease-causing pathogenic bacteria (Fig. 3). For instance, a specific strain of *Staphylococcus hominis* coagulase-negative staphylococci (CoNS), capable of producing autoinducing peptides that suppress toxin production by *S. aureus* through a quorum-sensing system, was used to decrease *S. aureus* survival and skin inflammation, without adverse effects on other skin commensals or the host in mice and human [139, 140]. *S. epidermidis* also plays a role in pathogen protection by producing AMPs and promoting AMPs secretion from the host [35, 82, 88–90]. Most attention in the dermatology field is paid to AD, and its treatment using live bacteria in human was first reported in 2018 [133]. *Roseomonas mucosa*, a gram-negative commensal bacterium, was isolated from the skin of healthy donors and was associated with improvement in pediatric AD severity, decreased *S. aureus* abundance, and reduced topical steroid requirements without severe adverse events [133, 141]. Moreover, skin improvements and colonization by the administered *R. mucosa* persisted for up to 8 months after cessation of treatment [141]. Autologous bacteriotherapy using CoNS, which is isolated from the non-lesional skin of each AD patient and kills *S. aureus* by producing AMPs, is thought to be safe and effective for AD patients with *S. aureus* overgrowth [142]. In addition to AD, treatments using live bacteria have been developed for acne vulgaris [143, 144]. A summary of treatment using live bacterial strains is presented in Table 1.

**Table 1:** Summary of bacteriotherapy using live bacterial strains for skin diseases

Reference	Experimental model	Target disease	Applied bacteria	Results
Myles et al. [133]	Human	AD	*Roseomonas mucosa*	Topical *R. mucosa* showed therapeutic activity against AD in adults and children without adverse events or treatment complications. (Decreases in disease severity, topical steroid requirement, and *S. aureus* burden)
Myles et al. [141]	Murine Human	AD	*Roseomonas mucosa*	*R. mucosa* application ameliorated disease severity of AD in children without severe adverse events. (Decreased disease severity, reduced *S. aureus* burden on the skin, and a reduction in topical steroid requirements, improvement in epithelial barrier function)
Nakatsuji et al. [78]	Murine Human	AD	Autologous bacterial transplant (*Staphylococcus epidermidis*, *Staphylococcus hominis*)	Reintroduction with antimicrobial strains of *S. epidermidis* or *S. hominis* decreased colonization density of *S. aureus* in AD patients
Williams et al. [139]	Murine	AD	*Staphylococcus hominis*	*S. hominis* inhibited *S. aureus* toxin production by quorum sensing and prevented *S. aureus*-mediated skin inflammation
Ito et al. [51]	Murine	AD	*Staphylococcus cohnii*	Colonization of *S. cohnii* ameliorated both AD-like dermatitis and psoriasis-like skin inflammation
Nakatsuji et al. [140]	Murine Human	AD	*Staphylococcus hominis*	Typical *S. hominis* A9 showed fewer adverse events and decreased *S. aureus* colonization in AD patients. (*S. hominis* A9 did not significantly improve disease severity but inhibited expression of toxin from *S. aureus*)
Nakatsuji et al. [142]	Human	AD	Allogeneic bacterial transplant (CoNS strains with antimicrobial activity)	Application of CoNS reduced *S. aureus* burden on lesional skin of AD patients without serious adverse events. (Decreased *S. aureus* burden, improved local disease severity score)
Karoglan et al. [143]	Human	Acne	*Cutibacterium acnes* from healthy individuals	Applied *C. acnes* reduced non-inflamed lesions (open and closed comedones) and skin pH without untoward adverse events
Lebeer et al. [144]	Human	Acne	*Lactobacilli* strains	Application of *Lactobacilli* strains reduced inflammatory lesions of acne. (Reduced relative abundance of staphylococci and *Cutibacterium acnes*)
Nakatsuji et al. [86]	Murine	Skin tumor	*Staphylococcus epidermidis*	*S. epidermidis* strain producing 6-N-hydroxyaminopurine (6-HAP) reduced the incidence of ultraviolet-induced tumors

*AD*, atopic dermatitis; *CoNS*, coagulase-negative Staphylococci

Since naturally occurring bacteriophages harbor specificity for a bacterial host, bacteriophage therapy is now receiving much attention in this era of antibiotic resistance [145]. Bacteriophage therapy has been a potential alternative antimicrobial strategy with a 100-year history of successful application without adverse effects [146]. The host specificity is remarkable, and inflammatory skin diseases such as *S. aureus*-induced AD-like dermatitis model [147] and acne [148] can be feasibly treated with bacteriophages. Moreover, bacteriophage therapy is also used for skin lesions in congenital diseases. Netherton syndrome, a rare autosomal recessive mutation in the serine protease inhibitor of Kazal-type 5 gene (*SPINK5*), is characterized by congenital ichthyosiform erythroderma, trichorrhexis invaginate, and atopic diathesis [21]. Treatment with several antistaphylococcal bacteriophages leads to an improvement in disease severity and substantial changes in symptoms [149]. Although the influence of bacteriophages on the entire microbial community needs to be carefully considered, they are expected to provide tailor-made antimicrobial therapies targeting specific pathogens.

**Fig. 4:**
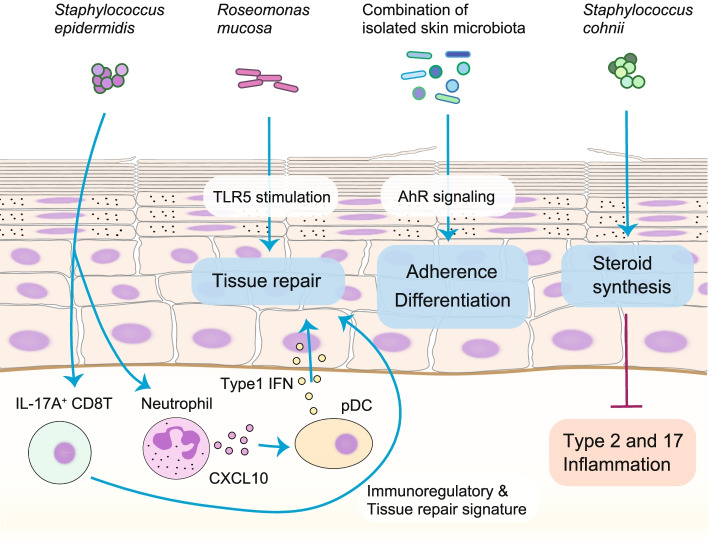
Potentially therapeutic host-microbe interactions. Colonization with specific skin commensals provides beneficial responses. *Staphylococcus epidermidis* stimulates immune cells such as lymphocytes and neutrophils to promote tissue repair. pDCs recruited by CXCL10 from neutrophils produce type-1 IFNs that activate tissue repair response. In parallel, IL-17A+ CD8T cells induced by *S. epidermidis* colonization show immunoregulatory and tissue repair signature genes. *Roseomonas mucosa* promotes tissue repair via Toll-like receptor 5 (TLR5) stimulation by tumor necrosis factor (TNF) signaling. The combination of isolated skin microbiota that is dominant in healthy human skin improves adherence and differentiation of keratinocytes through aryl hydrocarbon receptor (AhR) signaling. *Staphylococcus cohnii* promotes local steroid synthesis that suppresses types 2 and 17 skin inflammation

A body of evidence on bacteriotherapy is just beginning to emerge, and there may be many beneficial strains that can be used to treat skin diseases or maintain health (Fig. 4). *S. epidermidis* induces immune cells that promote tissue repair responses [82, 85]. Additionally, the application of *Roseomonas mucosa* promotes tissue repair through tumor necrosis factor signaling via TLR5 stimulation. The maturation of skin barrier function, such as proper differentiation and repair of the epidermal barrier, is mediated by members of the cutaneous microbiota that predominate in healthy human skin via the aryl hydrocarbon receptor on keratinocytes [51, 79]. *Staphylococcus cohnii* promotes local steroid synthesis and suppresses both types 2 and 17 response-induced dermatitis [51]. Therefore, manipulating the skin microbiota with specific bacterial strains will provide a new therapeutic option for maintaining homeostasis and improving diseased conditions not related to pathogenic bacteria. The skin microbiome is of growing interest in cosmetics, with a focus on using these properties to improve human health and well-being through formulations containing prebiotics and probiotics [60]. Further understanding of host-microbe interactions may lead to the development of bacteriotherapy, including administration of a tailor-made microbial cocktail that replenishes the deficient functions of human skin and assists in maintaining homeostasis in individuals.

#### Advantages of bacteriotherapy

The use of live bacteria has several potential advantages over the administration of exogenously purified chemical compounds. For instance, live bacteria can colonize specific locations in the body and deliver molecules at physiological concentrations directly to the host. This avoids redundant, high chemical doses, and off-target effects. Moreover, colonization with inoculated beneficial bacteria can be achieved, and microbial therapies have a long-lasting effect compared to the routine administration of purified molecules [141]. Finally, live bacteria stimulate multiple signaling pathways, some of which may be therapeutically useful but are yet to be determined in the host. Since host-microbe interactions activated by pathogen-associated molecular patterns are considered essential, treatment with purified bacterial metabolites may result in a loss of efficacy.

#### Disadvantages of bacteriotherapy

Although treatment with live bacteria can be a new and effective option for various diseases, it has some disadvantages. One of the notable concerns is safety and toxicity; as described above, even mutualistic bacteria that are not virulent in a homeostatic environment can cause local infection and bacteremia in some cases [150]. Therefore, it is necessary to ensure the safety of administered bacteria. Additionally, the stability of live bacteria and bacterial colonization is important. Repeated administration of live bacteria may be effective, even if they do not colonize the host; however, stable colonization should be achieved for long-term therapeutic effects [141]. Since the patient’s skin and other organs are also occupied by commensals, obtaining stable colonization with administered bacteria is possibly difficult by simple routine administration of live bacteria [135]. In addition, host immunity facilitates or inhibits colonization of transplanted microbial communities. To achieve stable colonization, a suitable administration method, such as pretreatment with antibiotics to open the niches for each bacterial cocktail, must be further refined [60]. Moreover, the development of optimal mediators to deliver live bacteria to their appropriate niches (for instance, sebaceous glands or HFs) is desirable for enhancing the therapeutic effect. As mentioned above, live bacteria activate various signaling pathways in the host; however, purified molecules may be more therapeutically useful when the activation of specific signaling pathways is not required.

## Conclusion

The impact of the skin microbial community on host physiology is an attractive target for therapeutic intervention, as the administration or elimination of particular microorganisms can considerably influence host conditions. In the new treatment concept of using the skin microbiota, there is still room for consideration of its safety and the administration of microbial strains. Expanding our understanding of how microbial communities influence host metabolic and immunological conditions will substantially increase our ability to rationally design bacteriotherapies.

## Data Availability

Not applicable

## Abbreviations

AD: Atopic dermatitis
AMP: Antimicrobial peptide
HF: Hair follicle
rRNA: Ribosomal RNA
ITS1: Internal transcribed spacer 1
TLR: Toll-like receptor
PSM: Phenol-soluble modulin
FMT: Fecal microbial transplantation
FDA: US Food and Drug Administration
CoNS: Coagulase-negative staphylococci
